# Expanding the Set of Three-Input Logic Functions in Inverted T-Shaped TFETs

**DOI:** 10.3390/mi14020445

**Published:** 2023-02-14

**Authors:** Hao Ye, Pengjun Wang, Gang Li, Yijian Shi, Bo Chen, Xiangyu Li, Jianping Hu

**Affiliations:** 1 College of Electrical and Electronic engineering, Wenzhou University, Wenzhou 325000, China; 2 College of Electrical Engineering and Computer Sciences, Ningbo University, Ningbo 315211, China

**Keywords:** tunneling field-effect transistors, three-input logic function, TCAD, compact logic gate

## Abstract

Three-input logic primitives show high efficiency in logic synthesis compared to traditional two-input logic, which encourages researchers to implement three-input logic gates with emerging nanotechnologies. This paper demonstrates a compact implementation of three-input monotone logic gates based on the inverted T-shaped TFET. Firstly, based on the gate coupling mechanism in the novel inverted T channel, the BTBT current can be suppressed in the vertical or horizontal region to achieve the channel strobe. Therefore, the typical three-input monotone logic functions, Majority, OrAnd, and AndOr, are successfully implemented on a single transistor. Then, a simplified potential model describing gate coupling is established to describe the impact of key device parameters on the logic behavior. Combined with TCAD simulation, the design rules of devices with different logic functions are given. Finally, a series of three-input monotonic logic gates are designed and verified. The results show that the use of the proposed TFETs can effectively save the number of transistors in the three-input logic gate, which indicates that the three-input TFET is a compact and flexible candidate for three-input logic gates.

## 1. Introduction

The beyond CMOS transistors with sub-60 mV/dec subthreshold swing SS for ultralow power applications are exploring alternatives, such as tunneling FET, negative capacitance FET and impact ionization FET [1,2,3,4]. Tunneling field-effect transistors (TFETs) with band-to-band tunneling (BTBT) as the main transmission mechanism can achieve a steeper SS. Because its manufacturing process is compatible with standard CMOS process platforms, it is considered one of the best candidates for next-generation logic devices [3,4]. Although silicon-based TFETs are confronted with problems such as weak driving capability and the parasitic bipolar effects, it is also actively studied and used to implement the logic gate with interesting characteristics [5,6,7,8,9,10,11,12,13,14]. Referring to [5,6], a set of hybrid TFET/CMOS logic families and topologies can achieve lower hardware costs and intrinsic delays than CMOS. A single double-gate TFET mentioned in [10,11,12,13,14] can exhibit two-input Boolean logic behaviors, such as Or, And, Or-not, and And-not, so it can achieve two-input logic gates compactly by reducing the number of transistors. For example, the NAND2 and NOR2 logic gates in [10,13] only consist of two TFETs, while four transistors are required for the conventional CMOS. A novel split-gate FET has been used as a single transistor AND gate with a lesser area and power dissipation for two-input logic implementation [15]. In contrast to two-input logic primitives, some three-input logic gates exhibit higher efficiency in logic network simplification due to their high expressive power, such as typical three-input monotonic logic, Majority (MAJ) gate, AndOr gate, OrAnd gate, et al. [16,17,18]. Researchers are using emerging nanotechnology to implement three-input logic gates, such as Mach-Zehnder interferometers (MZI), quantum cellular automata (QCA), single electron transistor (SET), and naphthalene-based single-molecule transistor (NST) [19,20,21,22,23]. Among them, the MZI can implement three-input logic gates in optical but it needs a complex photoelectric conversion. The QCA is limited by the high fault rate in IC fabrication. The NST and SET use a resistor instead of pull-up networks, resulting in a larger area and static power consumption. As for the TFET, there is still room for improvement and optimization to construct the above three-input logic gate circuit by using the double-gate TFET technology mentioned in [10,13] to save at least half of the number of transistors. Consequently, this paper will explore the use of fewer transistors to implement a series of three-input monotone logic gates. Therefore, the three-input TFETs for realizing the MAJ, AndOr, and OrAnd logic functions are proposed. Due to the interesting characteristics of the proposed transistor, we also demonstrate a flexible and compact physical implementation of the three-input logic gate by using proposed TFETs.

This paper is organized as follows. In the second part, the device structure and simulation method are described, and the manufacturing process for the proposed device is clarified theoretically. The third section investigates the coupling effect and establishes a simplified potential model. A method for realizing a three-input logic function by a single three-input TFET device is described in the fourth section. In the fifth section, the compact implementation of MAJ, AndOr, and OrAnd gates using the proposed TFET is demonstrated. Finally, a summary and expectation are given.

## 2. Device Structure and Simulation Configuration

**Figure 1:**
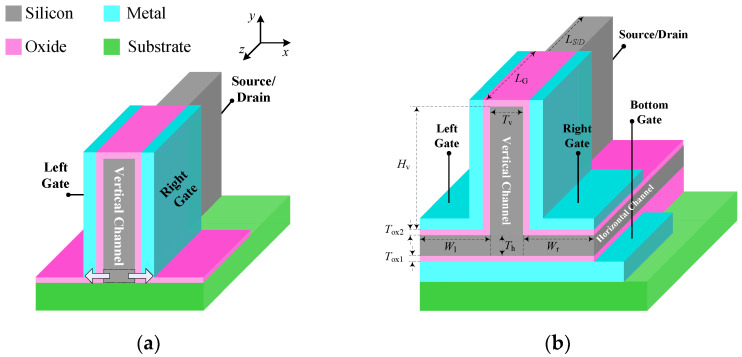
The three-dimensional structure diagram of the proposed three-input TFET. (**a**) DG-TFET. (**b**) Inverted T-shaped TFET.

The three-dimensional structure of the proposed three-input TFET device is illustrated in Figure 1b, where the channel is shaped like an inverted letter “T”. Unlike the two-input TFET shown in Figure 1a, in the region where the horizontal channel is formed, the etch does not extend down to the oxide, and thus the Fin can be integrated into both the horizontal and vertical directions of a novel inverted T-shaped channel. Two independently biased gates are located on both sides of vertical channel, and the bottom gate is buried under the horizontal channel.

The proposed TFET utilizes three independently biased gates in order to provide unconventional control of the channel electrostatic. Three independent gate terminals accept the logical input signal of “*ABC*”, “*A*” for the left gate, “*B*” for the right gate, and “*C*” for the bottom gate. By choosing the appropriate body thickness (*T*_h_, *T*_v_), gate work function *ϕ* (*ϕ*_L_, *ϕ*_R_, and *ϕ*_B_ are for left, right, and bottom gate), and the source doping level (*N*_S_h_, *N*_S_v_) of the horizontal and vertical regions, the BTBT in different biasing conditions can be suppressed. The remaining device parameters are listed in Table 1.

In this paper, we have employed Sentaurus TCAD software to predict the device properties. The dynamic nonlocal BTBT model is used in a 3D simulation environment to accurately describe the quantum tunneling current in a nonuniform electric field, where three important parameters in Nonlocal BTBT model, *p* = 2.5 for phonon-assisted tunneling, *A*_Si_ = 1.64 × 10^15^ cm^−3^·s^−1^, and *B*_Si_ = 23.8 × 10^6^ V/cm referred from [24]. Considering that the energy band distribution in the diffusion region is affected by the heavy doping, the band gap narrow model is activated to describe the change in the band gap. We also utilize the Shockley–Read–Hall recombination model to simulate the leakage currents due to thermal injection. In addition, a modified local density approximation model is used to estimate the confined carrier distribution in the thin-body channel. Furthermore, the effective electron mass and hole mass are 0.26 m0 and 0.36 m0 for Si semiconductor, respectively. The carrier transport directions are aligned to the <1 0 0> crystallographic directions in simulations.

**Figure 2:**
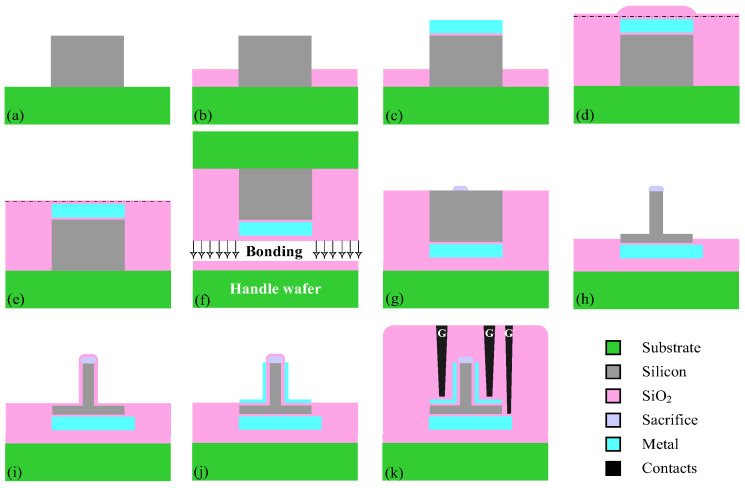
The manufacturing process flow of an inverted T-shape structure. (**a**–**c**) Fin process. (**d**) oxide deposition, (**e**) CMP, (**d**) Bonding, (**g**–**j**) selective etch and metal gate deposition, and (**k**) contacts connection.

The basic process flow of the three-input TFET is shown in Figure 2. The key fabrication process of the proposed device is compatible with the SOI platform, except that the same e-beam marks are employed before and after the molecular bonding to align the three independently biased gates [25]. It started with the growth of epitaxial silicon on the substrate, followed by selective etching of the epitaxial silicon. Subsequently, the oxide isolation, the gate oxide, and the metal are deposited sequentially, and then, the bottom gate is patterned. After that, the oxide is deposited on the substrate, flattened using CMP technology, and an oxide cladding layer is formed. Next, the wafer is bonded on a handle wafer so that the bottom gate is buried below the channel, and as the next step, the inverted T-shape silicon is formed where the selective etch does not extend to the oxide. Finally, after the deposition of the SiO_2_, the left/right gate, the source, gate, and drain contacts are connected.

## 3. Gate Coupling in the Three-Input TFET

For a single device to exhibit MAJ (*F* = *AB* + *BC* + *CA*), OrAnd (*F*= *AC* + *BC*), and AndOr (*F* = *AB* + *C*) logic behavior, the challenge is to inhibit the on-state current when only one logic “1” is input (e.g., the N-type device should be in the cut-off state, where *ABC* = “100”, and the drain current is expressed as *I*_001_), which is diametrically opposed to the characteristics of a conventional MOSFET. The on-state current of TFET is mainly contributed by BTBT, and the BTBT current is most sensitive to the potential variation. Unlike the conventional TFET, the potential distribution in the inverted T-shaped channel is jointly modulated by the three gates, so the study of the coupling effect between the gates will help to find the appropriate device parameters to design a three-input TFET.

**Figure 3:**
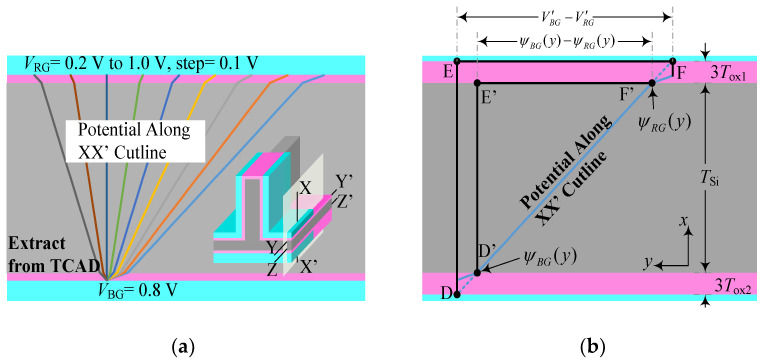
(**a**) Potential distribution (extracted by TCAD) along the XX’ cutline for increasing *V*_RG_ from 0.2 V to 1.0 V in steps of 0.1 V, where *V*_BG_ is fixed at 0.8 V. (**b**) Simplified model of the potential along the XX’ cutline. All cutlines lie in the YZ plane, and the YY’ and ZZ’ cutlines indicate the surface potential near the right and bottom gates, respectively.

The potential distribution of the inverted T-shape channel for increasing *V*_RG_ from 0.2 V to 1.0 V in steps of 0.1 V, where *V*_BG_ is fixed at 0.8 V, is shown in Figure 3a. The important properties are visible: (1) The electrostatic potential in the right region of the horizontal channel is modulated by *V*_RG_ and *V*_BG_. (2) The potential along XX’ cutline is weakly affected by *V*_DS_ and presents a straight distribution. 

To investigate the coupling effect on the conductive characteristics, a surface potential can be derived as a compact function of the body thickness *T*_Si_ and the oxide thickness *T*_ox_. If *ε*Si = *ε*SiO_2_, the potential along the XX’ cutline will not bend at the Si-SiO_2_ interface. In order to obtain the same equivalent thickness of oxide, the *T*_ox_ can be increased by *ε*Si/*ε*SiO_2_ = 11.9/3.9 ≈ 3 times [26]. Since the device is a long channel device, the potential distribution along the XX’ line is not affected by the source/drain potential. Therefore, the potential distribution shown in Figure 3b is linear, which is more convenient for intuitive analysis. 

**Figure 4:**
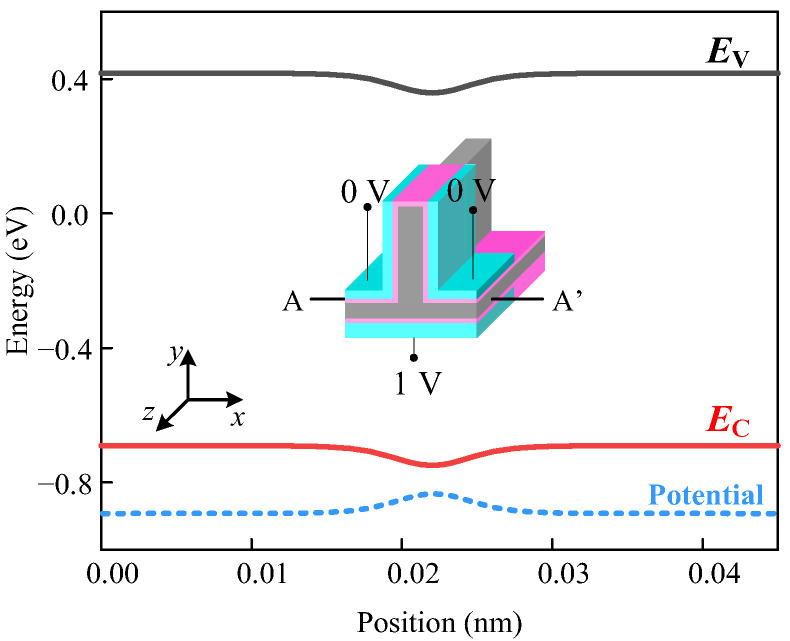
The potential and energy band distributions along cutline AA’ in x-axis.

The potential distribution and energy band distributions along the x-axis are obtained by TCAD tools shown in Figure 4, where the input logic of ABC equals “001”. As we can see, the potential and energy band along the x-axis changes smoothly, and the current flow direction is perpendicular to the x-axis (in the yz-plane). Therefore, it is worth mentioning that we assumed that the carriers only flowed perpendicular to the x-axis, and thus only analyzes the potential distribution in the yz-plane.

Using the above simplified approach, the potential distribution (*V*_RG_ = 1 V, *V*_BG_ = 0 V) can be depicted as a straight line DF as shown in Figure 3b, where two similar triangles DEF and D’E’F’ can be obtained. Therefore, the simplified analytical model can be expressed as:

(1) $$\frac{{V^{\prime }}_{\mathrm{RG}}-{V^{\prime }}_{\mathrm{BG}}}{\psi_{\mathrm{RG}}(x)-\psi_{\mathrm{BG}}(x)}=\frac{3(T_{\mathrm{ox}1}+T_{\mathrm{ox}2})+T_{\mathrm{Si}}}{T_{\mathrm{Si}}}$$

According to (1), *ψ*_RG_(*x*) and *ψ*_RG_(*x*) represent the surface potential near the right gate and the bottom gate, respectively. ${V^{\prime }}_{\mathrm{RG}}$ and ${V^{\prime }}_{\mathrm{BG}}$ are the difference between the gate voltage and the flat-band voltage, as given by ${V^{\prime }}_{\mathrm{RG}}=V_{\mathrm{RG}}-V_{\mathrm{RG}\_\mathrm{fb}}$ and ${V^{\prime }}_{\mathrm{BG}}=V_{\mathrm{BG}}-V_{\mathrm{BG}\_\mathrm{fb}}$. Equation (1) can be rewritten as:

(2) $$\Delta \psi =\psi_{\mathrm{RG}}(x)-\psi_{\mathrm{BG}}(x)=\frac{V_{\mathrm{RG}}-V_{\mathrm{BG}}-\Delta \varphi }{1+\frac{3(T_{\mathrm{ox}1}+T_{\mathrm{ox}2})}{T_{\mathrm{Si}}}}$$

where Δ*ϕ* is the difference between the work functions of the right gate and bottom gates. To simplify the analysis, take *T*_ox1_ and *T*_ox2_ as 1 nm, and Δ*ϕ* = 0 eV. Equation (2) can be rewritten as:

(3) $$\Delta \psi =\frac{V_{\mathrm{RG}}-V_{\mathrm{BG}}}{\frac{6}{T_{\mathrm{Si}}}+1}$$

The relationship between the body thickness and the surface potential difference Δ*ψ* is plotted in Figure 4a, where *V*_LG_ = *V*_RG_ = 0 V, *V*_BG_ = 1 V. We observed that the |*q*·Δ*ψ*| increases as body thickness increases and converges to the difference between *V*_RG_ and *V*_BG_ (*V*_BG_ − *V*_RG_ = 1 V). The surface potential on each side is modulated by the two gate voltages simultaneously. If the coupling is weaker, the |*q*·Δ*ψ*| is closer to *V*_BG_ − *V*_RG_. Conversely, the potential difference is much smaller than *V*_BG_ − *V*_RG_.

**Figure 5:**
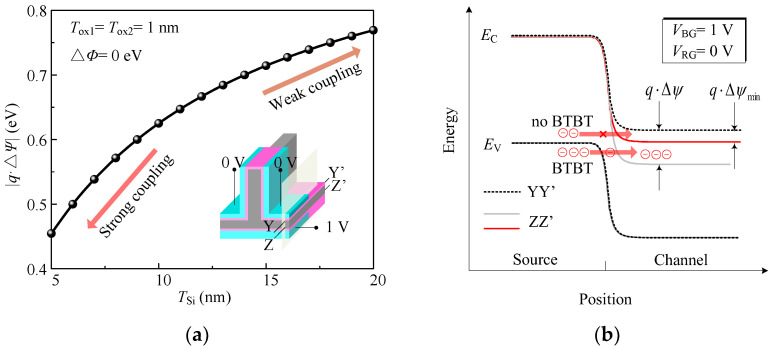
(**a**) The variation of the |*q*·Δ*ψ*| with the various body thickness at input logic of *ABC* = “001”. (**b**) Energy band distribution along YY’ and ZZ’ cutline.

Taking inhibition of *I*_001_ as an example, where *V*_LG_ = *V*_RG_ = 0 V and *V*_BG_ = 1 V. We assume that the *ϕ* of the right gate is large, which causes no BTBT occurring near the right gate, and the energy band distribution along the YY’ cutline is shown as the dashed line in Figure 5b. We find that the |*q*·Δ*ψ*| is too large, and the BTBT will occur along the ZZ’ cutline resulting in high *I*_001_ in this condition, as shown by gray solid lines in Figure 5b. Referring to (3), it can be seen that reducing the *T*_Si_ can reduce the |*q*·Δ*ψ*|. If the coupling is small enough to make |*q*·Δ*ψ*|<|*q*·Δ*ψ*_min_|, the conduction band energy in the channel will be higher than the valence band in the source region, as shown by red solid line in Figure 5b. Therefore, the BTBT will not occur, and the purpose of suppressing the *I*_001_ is achieved.

This section may be divided by subheadings. It should provide a concise and precise description of the experimental results, their interpretation, as well as the experimental conclusions that can be drawn.

## 4. Extending the Logic Behavior of a Single TFET Device

### 4.1. Majority Behavior

An N-type device exhibiting MAJ logic behavior is turned on in which at least two of gate biases of 1 V. Therefore, the drain current, such as *I*_100_, *I*_010_, and *I*_001_, is needed to be suppressed in order to obtain a high *I*_on_/*I*_off_ ratio. From the analysis in the previous section, two important device parameters should be concerned: (1) A large work function *ϕ* (*ϕ*_L_, *ϕ*_R_, *ϕ*_B_). (2) A thin channel (*T*_h_, *T*_v_) to obtain a stronger coupling. The device parameters for the other devices are also given in Table 2.

**Figure 6:**
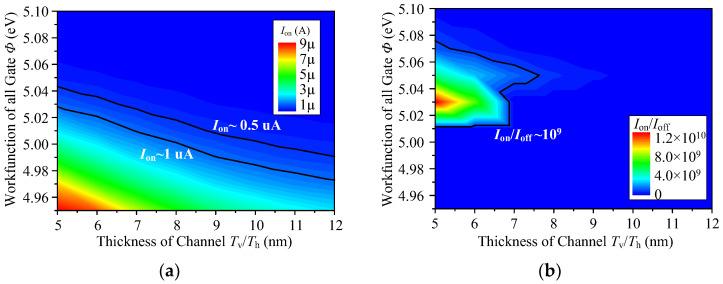
(**a**) The on-state current *I*_on_ versus work function *ϕ* and body thickness *T*_h_/*T*_v_. (**b**) The *I*_on_/*I*_off_ ratio versus *ϕ* and *T*_h_/*T*_v_.

As verified by TCAD, the best device performance is achieved with the *ϕ* set to 5.03 eV for three gates and the *T*_h_/*T*_v_ set to 5–7 nm, as shown in Figure 6. It is worth noting that the proposed device is sensitive to the fluctuation of the *ϕ*; thus, requires a strict process control for the gate.

**Figure 7:**
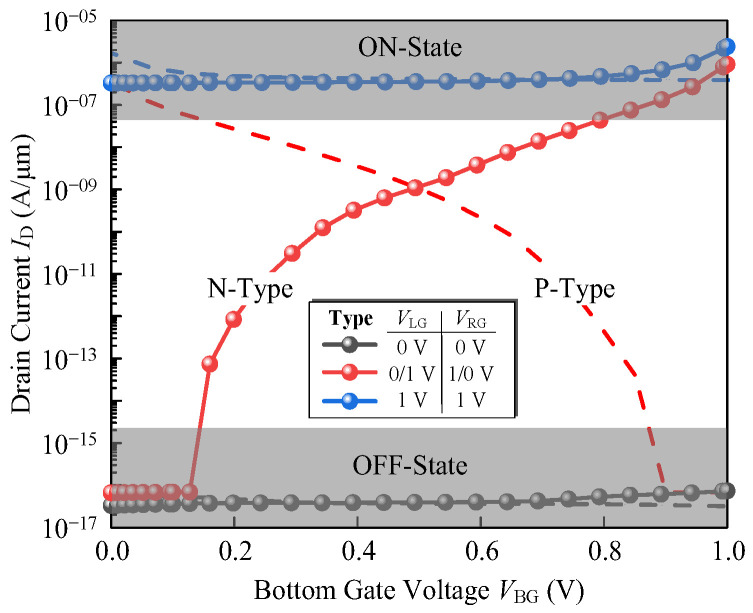
*I*-*V* characteristics of the N-type and P-type MAJ logic transistor at *V*_DS_ = 1 V.

The transfer characteristics of the three-input TFET are obtained by scanning the bottom gate, where the *V*_LG_ and *V*_RG_ are fixed at logic “00”, “01”, “10” or “11”, as shown by the dotted lines in Figure 7. As can be seen, a single transistor exhibits MAJ logic behavior. When the *V*_LG_ and *V*_RG_ are both fixed at 0 V, the device is always turned off regardless of the variation of *V*_BG_. In contrast, the device is in on-state at *V*_LG_ = *V*_RG_ = 1 V. The *I*_on_/*I*_off_ ratio above ~10^9^ are obtained, as well as the low turn-off current *I*_off_. However, the on-state current is still small, which is a common disadvantage for silicon-based TFETs but meets the requirement of the minimum current of 1 µA for low-power transistors as indicated by IRDS2020 [27].

**Figure 8:**
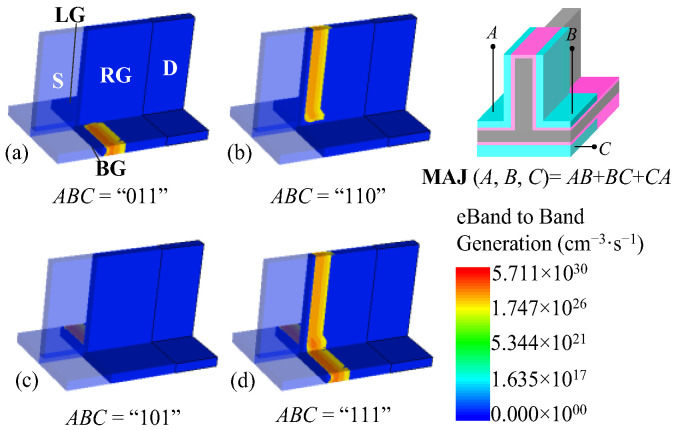
Schematic diagram of BTBT generation with different gate inputs in MAJ logic device. (**a**) *ABC* = “011”, (**b**) *ABC* = “110”, (**c**) *ABC* = “101”, and (**d**) *ABC* = “111”.

The BTBT generation distribution in the inverted T channel at different gate inputs reveals different on-state mechanisms, as shown in Figure 8. Taking the condition of *ABC* = “110” as an example (Figure 8b), the BTBT only occurs in the vertical channel, and thus the drain current flows through the vertical region. Due to the strong coupling, the BTBT in the horizontal channel is suppressed, resulting in an almost zero BTBT generation rate in this region. The different gate inputs lead to distribution differences of the BTBT, which shows the channel strobe mechanisms of the proposed device.

To build a static complementary MAJ logic gate, a P-type TFET with MAJ behavior is also indispensable. The design of a P-type TFET is similar to an N-type TFET with reversed doping type. The doping concentrations of the N-doped source and P-doped drain are 1 × 10^20^ cm^−3^ and 4 × 10^18^ cm^−3^. The *I*-*V* characteristics of the P-type device of *ϕ* = 4.22 eV are shown as dashed lines in Figure 7, which shows a good symmetry.

### 4.2. OrAnd Behavior

Comparing the MAJ (*F* = *AB* + *AC* + *BC*) and OrAnd (*F* = *AC* + *BC*) functions, the OrAnd logic function lacks the *AB* term. Therefore, suppressing the drain current *I*_110_ in the vertical channel at the gate inputs of *AB* = “11” is also one of the keys in an OrAnd logic device. According to the analysis in Section 3, this can be achieved by appropriately increasing the *T*_v_ to reduce the coupling. The other device parameters for the OrAnd device are shown in Table 2.

**Figure 9:**
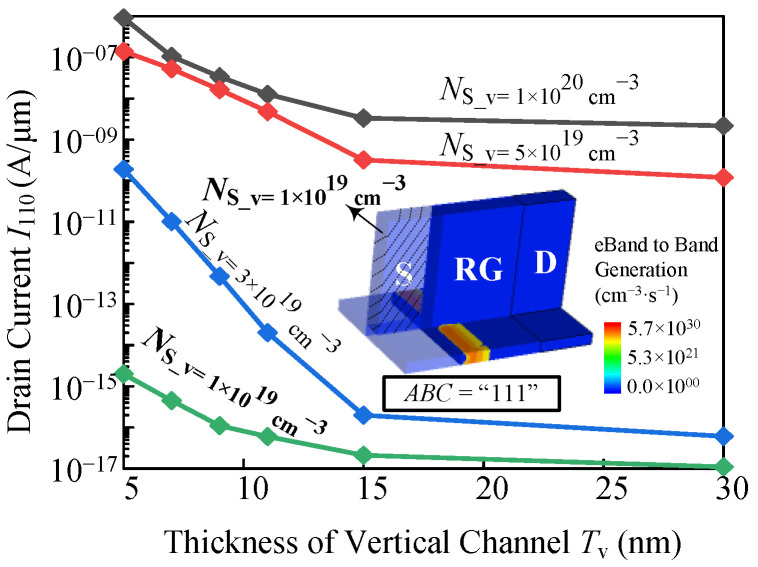
The *T*_v_ varied with *I*_110_ at *N*_S_v_ = 1 × 10^20^ cm^−3^, 5 × 10^19^ cm^−3^, 3 × 10^19^ cm^−3^, and 1 × 10^19^ cm^−3^ in the OrAnd logic device.

The black curve in Figure 9 indicates the *I*_110_ with various *T*_v_. As *T*_v_ increases from 5 nm to 30 nm, the *I*_110_ decreases from 1.02 × 10^−6^ A/μm to 2.18 × 10^−9^ A/μm. The decreasing trend of the *I*_110_ slows down when the *T*_v_ is above 15 nm, which is highly consistent with the tendency in Figure 5a. However, the *I*_110_ is still high, which will lead to the large leakage power. Considering the low leakage and the simple process, the doping concentration of the vertical source region is chosen to be optimized. Therefore, we explored the device performance with different *N*_S_v_ and found the appropriate value. As shown in Figure 9, for a device with *N*_S_v_ = 1 × 10^19^ cm^−3^ and *T*_v_ = 9 nm, the *I*_110_ is suppressed to less than 10^−16^ A/μm. The inset of Figure 9 shows the contour plot of the BTBT generation rate at *ABC* = “111”, from which we observe that no BTBT occurs in the vertical channel and the drain current only flows through the horizontal region, even for *V*_LG_ = *V*_RG_ = 1 V.

Considering the current characteristics in all on-state of OrAnd devices (no BTBT current in vertical channel), complete removal of the vertical fin would be a better option. In order to obtain the same structure of all logic transistors, the doping concentration of the vertical source region is chosen to be optimized. However, ion implantation with different implantation dose and ionic energy is required for vertical and horizontal channels, resulting in additional shading layers over vertical and horizontal channels and extra process steps.

**Figure 10:**
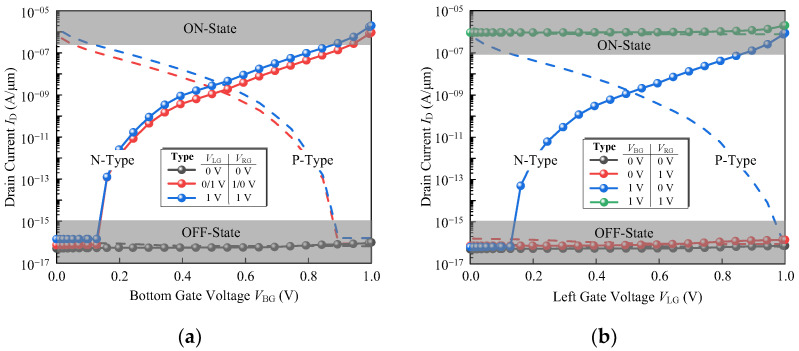
(**a**) *I*_D_-*V*_BG_ and (**b**) *I*_D_-*V*_LG_ curve of the proposed device exhibiting OrAnd behavior. The dotted lines are for N-type device and dashed lines for P-type.

Without increasing the device width (*W*_l_ + *W*_r_ + *T*_v_), the smaller the *T*_v_ the larger the effective area of the horizontal channel is obtained. Therefore, the *T*_v_ is taken as 9 nm, and the *I*_D_-*V*_BG_ and *I*_D_-*V*_LG_ characteristics of the N-type device are shown in Figure 10 (dotted lines). For a P-type TFET, the vertical source is 1 × 10^19^ cm^−3^ N-doped, the horizontal source is 1 × 10^20^ cm^−3^ N-doped, and the drain is 1 × 10^18^ cm^−3^ P-doped. The transfer characteristics of the P-type OrAnd logic device at *ϕ* = 4.22 eV are shown in Figure 10 (dashed lines).

### 4.3. AndOr Behavior

The pull-up network of an OrAnd logic gate needs to exhibit AndOr logic behavior, so a three-input TFET with the AndOr logic behavior is needed to be realized. For an AndOr logic device, only the BTBT current in the vertical channel at gate inputs of *AB* = “10” or *AB* = “01” needs to be suppressed. Consequently, the device parameters related to the vertical channel can remain unchanged, such as the *ϕ* of the left and right gate and *T*_v_. It is worth noting that the device is turned on at *ABC* = “001”, so the coupling in the horizontal should not be enhanced.

**Figure 11:**
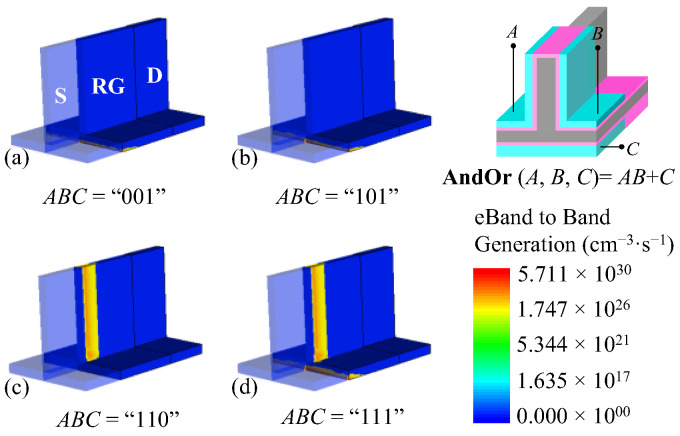
Contour plot of the BTBT generation rate for different gate inputs in AndOr logic device. (**a**) *ABC* = “001”, (**b**) *ABC* = “101”, (**c**) *ABC* = “110”, and (**d**) *ABC* = “111”.

On the basis of the MAJ logic device, the *ϕ* of the bottom gate is reduced to 4.2 eV, and the *N*_S_v_ is set to N-doped 5 × 10^19^ cm^−3^. The BTBT generation rate distribution under different biasing condition is obtained, as shown in Figure 11. As we can see, the BTBT in the horizontal channel is modulated by the *V*_BG_ only, and the BTBT only occurs near the bottom region in the horizontal channel due to the small threshold voltage of the bottom gate.

**Figure 12:**
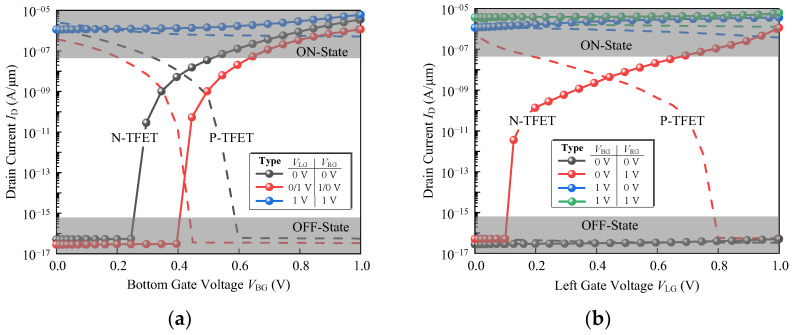
(**a**) *I*_D_-*V*_BG_ and (**b**) *I*_D_-*V*_LG_ curve of the proposed device exhibiting AndOr behavior. The dotted lines are for N-type device and dashed lines for P-type.

The *I*_D_-*V*_BG_ and *I*_D_-*V*_LG_ characteristics of the N-type (dotted lines) and P-type (dashed lines) device are shown in Figure 12. For the P-type AndOr device, the *ϕ* of the left and right gates are both set to 4.22 eV and 5.1 eV for the bottom gate.

## 5. Discussion

**Figure 13:**
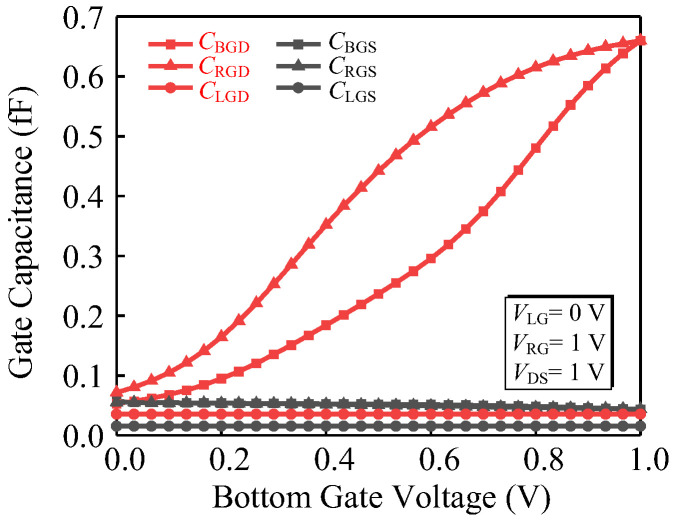
Capacitance characteristics of the proposed device. *C*_BGS_, *C*_RGS_, and *C*_LGS_ indicate the gate-source capacitance, and *C*_BGD_, *C*_RGD_, and *C*_LGD_ indicate the gate–drain capacitance.

After optimizing the device parameters, critical electrical performances are summarized in Table 3, demonstrating correct logic behavior with *I*_on_/*I*_off_ ratio of more than 10^−9^. We also applied a small signal voltage of 1 MHz to the proposed device and extracted the parasitic capacitance curve, as shown in Figure 13. For the three-input TFET, the inversion layer is only connected to the drain, resulting in the gate–drain capacitance constituting a higher fraction of gate–gate capacitance *C*_gg_ [28]. Since the *V*_RG_ is fixed at 1 V, the inversion first occurs in the horizontal channel near the right gate, with the right gate–drain capacitance *C*_RGD_ accounting for a large proportion of *C*_gg_. When the *V*_BG_ is scanned to 1 V, the bottom gate–drain capacitance *C*_BGD_ and *C*_RGD_ are equal. The TFETs have a large Miller capacitance (*C*_GD_) compared to MOSFET; thus, obtain the larger overshoot resulting in a degradation of switching characteristics. Referring to [28], the *C*_GD_ can be reduced through design improvements made to the drain dopant profile or through *L*_g_ scaling.

**Figure 14:**
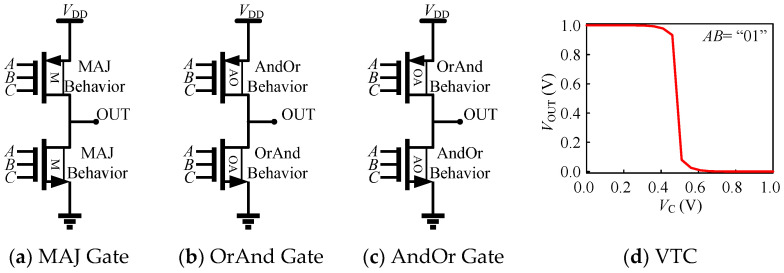
(**a**–**c**) Construction of compact MAJ gate, OrAnd gate, and AndOr gate based on three-input TFETs. (**d**) VTC characteristic curve of MAJ gate, where *AB* = “01”.

To implement static complementary three-input logic gates, we divide the six logic devices into three complementary pull-up and pull-down transistor pairs, such as the pair of P-type MAJ and N-type MAJ logic TFETs, the pair of P-type OrAnd and N-type AndOr logic TFETs, and the pair of P-type AndOr and N-type OrAnd logic TFETs. Figure 14a–c illustrates the compact implementation of the three logic gates, as we can see, using the proposed transistors to realize three-input logic gates, which can greatly save the number of transistors. The voltage transfer characteristics of the MAJ gate are shown in Figure 14d, which shows good static performance.

Using the delay estimation method of logic gates mentioned in [29] reveals that the three-input TFET logic gate still has a high delay compared to the conventional CMOS gates. It is due to the low on-state current and high gate-drain capacitance in silicon-based TFETs. However, it is worth mentioning that implementing three-input logic gates by using the proposed devices can significantly reduce the number of transistors. In addition, the use of proposed devices could reduce the load capacitance and stack height, which results in an improvement of high delay in TFET-based circuits.

If carriers are confined in the thin body, geometric quantization leads to an energy shift of *E*_C_ and *E*_V_ leading to a larger band gap and effective mass in proposed devices. Therefore, we plot the transfer characteristics for the MAJ logic device obtained by the semi-classical model and quantum confinement model. Compared to the semi-classical models, the quantum mechanical treatment reveals a shift of the onset of 130 mV and degradation of subthreshold swing.

**Figure 15:**
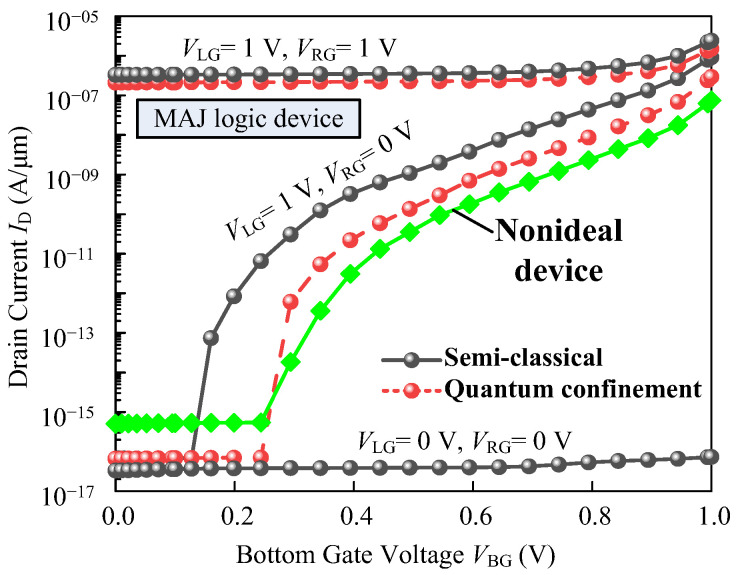
The transfer curves of the MAJ logic device extracted by TCAD simulation: (i) semi-classical, (ii) considering quantum confinement effect, and (iii) considering nonideal effects.

Furthermore, the second-order effects such as trap-assisted tunneling and non-homogenous doping are also taken into account. The nonideal transfer curve is shown in Figure 15, where the SS and *I*_on_/*I*_off_ ratio degradation is predicted but does not affect the logic behavior of the device. In general, degradation of device performance will cause degradation of proposed logic gate performance, such as increased static power consumption, increased delay, and low drive current.

**Figure 16:**
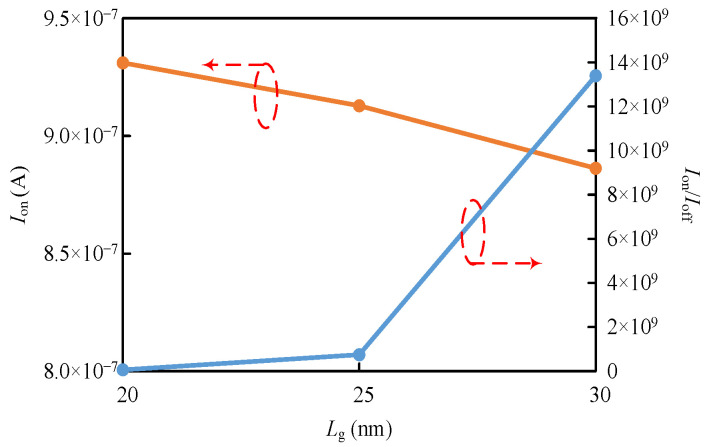
The *I*_on_ and *I*_on_/*I*_off_ of the MAJ logic device with same body thickness and oxide thickness.

Scaling of *L*_G_ from 30 nm to 20 nm is investigated, as shown in Figure 16. As *L*_G_ is scaled with the same body thickness and oxide thickness, *I*_on_ and *I*_on_/*I*_off_ degrades. To achieve the proposed MAJ/AndOr/OrAnd gate, a design with a thin channel and a small *T*_ox_ is required. This would be problematic for scaling. The tunneling current and *I*_on_/*I*_off_ of TFETs are very sensitive to body thickness, as shown in Figure 6. The sensitivity of TFET characteristics to oxide thickness is not significant [30]. Therefore, the main goal to recover degraded characteristics due to scaled-*L*_G_ is using a thinner body, which will have negative effects on the MAJ device process and device variability.

## 6. Conclusions

This paper presents a novel TFET with an inverted T channel structure that can compactly implement MAJ, OrAnd, and AndOr logic gates. Under the different biasing conditions, the channel strobe mechanism is achieved through suppressing the coupling in the horizontal or vertical channel. The impact of the body thickness, work function and doping concentration on logic behavior and device performance are studied in detail with the help of the proposed simplified model and TCAD. The results show that realize three-input logic gates by using the proposed devices can save the number of transistors, and has low load capacitance and leakage current. Compared to the traditional CMOS logic gates, silicon-based TFET gates still have the problem of high delay, and extending the logic behavior in the three-input TFET also has negative effects on the device process and device variability. However, the use of three-input TFETs will provide a flexible and compact candidate for physical implementation of three-input logic functions.

The main objective of this paper is to propose techniques for extending the three-input logic behavior in a single silicon-based TFET, rather than enhancing device performance. In the future, the use of narrow energy gap materials or heterojunction technology on the silicon-based three-input TFETs will lead to improved device performance.

## Figures and Tables

**Table 1:** The parameters of the proposed TFET.

Parameter	Value
Vertical Channel Height (*H*_v_)	40 nm
Gate Length (*L*_G_)	30 nm
Source/Drain Length (*L*_S_/*L*_D_)	30 nm
Gate Oxide Thickness (*T*_ox1_/*T*_ox2_)	3 nm
Left/Right Gate Width (*W*_l_/*W*_r_)	15 nm
Channel Doping Profile (*N*_C_)	1 × 10^17^ cm^−3^
Drain Doping Profile (*N*_D_)	1 × 10^18^ cm^−3^

**Table 2:** Key parameter configuration of three-input TFETs with different logic behaviors.

Type	*ϕ*_L_	*ϕ*_R_	*ϕ*_B_	*T*_h_	*T*_v_
MAJ	Large	Large	Large	Thin	Thin
OrAnd	Large	Large	Large	Thin	Thick
AndOr	Large	Large	Small	Thick	Thin

**Table 3:** Important electrical performances of the proposed devices and logic gates.

Logic Behavior	Type	*I*_on_ (A/μm)	*I*_on_/*I*_off_	Delay (ns)
MAJ	N-type	9.13 × 10^−7^	~10^10^	14.3
	P-type	6.34 × 10^−7^	~10^09^	
OrAnd	N-type	9.36 × 10^−7^	~10^10^	15.8
	P-type	7.57 × 10^−7^	~10^09^	
AndOr	N-type	1.17 × 10^−6^	~10^10^	9.2
	P-type	5.12 × 10^−7^	~10^10^	

## Data Availability

The data presented in this study are available on request from the corresponding author.
